# Diagnosis of Mucopolysaccharidosis Based on History and Clinical Features: Evidence from the Bajio Region of Mexico

**DOI:** 10.7759/cureus.3617

**Published:** 2018-11-20

**Authors:** Douglas Colmenares-Bonilla, Christian Colin-Gonzalez, Alejandra Gonzalez-Segoviano, Enrique Esquivel Garcia, Ma Martha Vela-Huerta, Fanny Guadalupe Lopez-Gomez

**Affiliations:** 1 Orthopaedics, Hospital Regional De Alta Especialidad Del Bajio, Leon, MEX; 2 Orthopaedics, Hospital General De Leon, Leon, MEX; 3 Orthopaedics, Hospital De Especialidades Pediatricas De Leon, Leon, MEX; 4 Orthopedics, Hospital General De Leon, Leon, MEX; 5 Pediatrics, Hospital General De San Luis De La Paz, San Luis de La Paz, MEX

**Keywords:** mucopolysaccharidosis, mexico, diagnosis, short stature, dwarfism, orphan disease, lysosomal disease

## Abstract

Introduction

Mucopolysaccharidosis (MPS) are infrequent deposit diseases; generally, the diagnosis is delayed until symptoms appear. Age or presentation is related to the severity of the disease. A substantial number of patients are misdiagnosed since they describe nonspecific initial symptoms and signs in common. The aim of this study is to describe the common characteristics of patients with mucopolysaccharidosis already diagnosed, treated in hospitals of the Guanajuato Health System, with a special focus on early manifestations in order to review early clinical suspect manifestations.

Methods

A multicenter, descriptive, observational study was conducted to evaluate the cases of mucopolysaccharidosis treated and diagnosed. The study was carried out in the Pediatric departments of five big important hospitals of Bajio Mexico region in the period from February to August 2016.

Results

Eighteen patients were identified, 13 men and five women, with an average age of 8.6 years. The most frequent mucopolysaccharidosis was type IV A (Morquio) in seven patients, followed by type I (Hurler) in four patients, three patients for type III (San Filippo), two patients for type II (Hunter), and two patients for type VI (Maroteaux-Lamie). The commonest clinical manifestations at diagnosis were dimorphism, triangular dorsal hump, skeletal alterations (genu valgus, short stature, and flat feet), and a limited range of movement in the major joints. Non-skeletal manifestations, such as an umbilical/inguinal hernia and hepato-splenomegaly, were very frequent. In a majority of patients with mucopolysaccharidosis, the radiological data of the disease were found: they were most severe in type IV and type VI, mild in type I and II, and none in MPS III. A diagnosis was made in all patients by a clinical and radiological evaluation and confirmed by an enzymatic study.

Conclusions

In all rare diseases, a suspicion diagnosis is based on subtle characteristics that manifest themselves in a few different organs and systems may be mild. Suspicion by the physician and the need to strengthen collaboration patterns between different specialities play an important role in the early diagnosis and treatment of these conditions.

## Introduction

The term mucopolysaccharidosis (MPS) groups a series of rare conditions, characterized by defects in the catabolism of glycosaminoglycans (GAGs) [1], which causes the progressive accumulation of these substances in cellular lysosomes on different tissues [2], resulting in pathologic progressive changes in multiple systems that will affect the quality of life in the short or medium term [3-5].

Generally, manifestations of these diseases will be related to the type of GAGs accumulating in cellular lysosomes. Accumulation is as follows: dermatan and heparan sulfate in the Hurler (MPS I) and Hunter (MPS II) syndromes; heparan sulfate in Sanfilippo syndrome (MPS III); keratan and chondroitin sulfate in Morquio syndrome (MPS IV A) [6]; and dermatan sulfate in Maroteaux-Lamy syndrome (MPS VI) [7-8].

Currently, there are seven described types of MPS, caused by the deficiency of 10 hydrolases [9]; these are mentioned in Table 1.

**Table 1:** Current classification of mucopolysaccharidosis, name, stored glycosaminoglycan, and enzymatic deficiency Name, stored glycosaminoglycan and enzymatic deficiency.

TYPE	NAME	STORED GLYCOSAMINOGLYCAN	ENZYME
I	Hurler	Heparan sulfate. Dermatan sulfate.	α-L-iduronidase
	Hurler-Scheie		
	Scheie		
II	Hunter.	Heparan sulfate. Dermatan sulfate.	Iduronate-2-sulfatase
III	San Filippo A	Heparan sulfate.	Heparan N-sulfatase
	San Filippo B		α-N-acetyl glucosaminidase
	San Filippo C		α-glucosaminide acetyltransferase
	San Filippo D		N-acetylglucosamine 6- sulfatase
IV	Morquio A	Keratan sulfate + Chondroitin-6-sulfate. Keratan sulfate.	N-acetylgalactosamine-6- Sulfatase
	Morquio B		β-D-galactosidase
VI	Maroteaux- Lamie.	Dermatan sulfate.	N-acetylgalactosamine-4- sulfatase
VII	Sly.	Heparan sulfate + Dermatan sulfate + Chondroitin-4, -6 sulfate.	β-glucuronidase
IX	-	Hyaluronan	Hyaluronidase

Diagnosis can represent a challenge, and an agreement between clinical and radiological findings is required. At birth, patients phenotypically have a normal appearance. The onset of the development of symptoms varies depending on the severity of the disease [10]; those severely affected show signs and symptoms in the first year of life while the attenuated forms do so in childhood or adolescence [11].

The age of first clinical signs in patients with a severe phenotype can be evidenced from birth, diagnostic suspicion, and referral to the specialist as early as four months; however, in attenuated patients, this suspicion may be delayed even after 10 or 12 years [12].

Diagnosis confirmed by an enzymatic study varies between 20 and 522 months of age, in some reports, with an average of 62 months [13-14].

**Figure 1:**
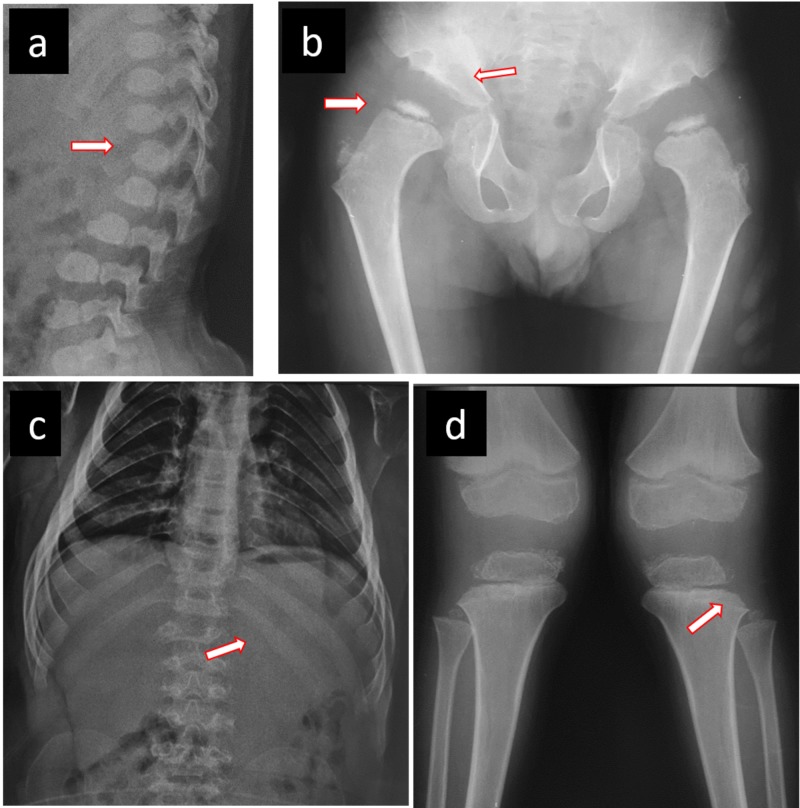
Radiographic features in an MPS IV A male patient a) Central beaking of the first lumbar vertebrae and posterior vertebral scalloping. b) Severe acetabular dysplasia, coxa plana, broad neck of femur. c) Flattened ribs, hepatosplenomegaly. d) Genu valgus with proximal and lateral tibial dysplasia, widening of the physeal line. MPS: mucopolysaccharidosis

Among the clinical findings found in MPS are general manifestations, such as short stature, infiltrated facies, thick eyebrows, as well as orthopedic manifestations (joint hypermobility or joint stiffness without inflammation, skeletal deformities, and multiple dysostosis), cardiac manifestations (murmur, myocardiopathy, mitral-aortic valve disease, cardiomegaly), and respiratory manifestations (hypertrophy of tonsils, adenoids, frequent respiratory infections). In other patients, there are umbilical and/or inguinal hernias, global retardation of psychomotor development, and the progressive loss of motor and intellectual skills they had already acquired in all but attenuated phenotypes and MPS IV and VI [3-4,6,10-11,13,15-16] (Figure 1).

Each type of glycosaminoglycan has major storage organs: heparan sulfate produces predominantly neurological symptoms, as in MPS I, II, and III; keratan sulfate produces corneal opacities and skeletal alterations without the neurological involvement observed in MPS IV A and VI; and dermatan sulfate produces cardiomyopathy and valvular heart disease in MPS I, II, and VI [5-6,10,13,17].

It is recommended to carry out a clinical and radiographic evaluation of different regions of the skeleton for patient assessment. These radiographs are cranial in the lateral view, complete lateral spine, anteroposterior pelvis, and extremities, particularly knees, and hands and wrists in anteroposterior views [10,18-20].

**Figure 2:**
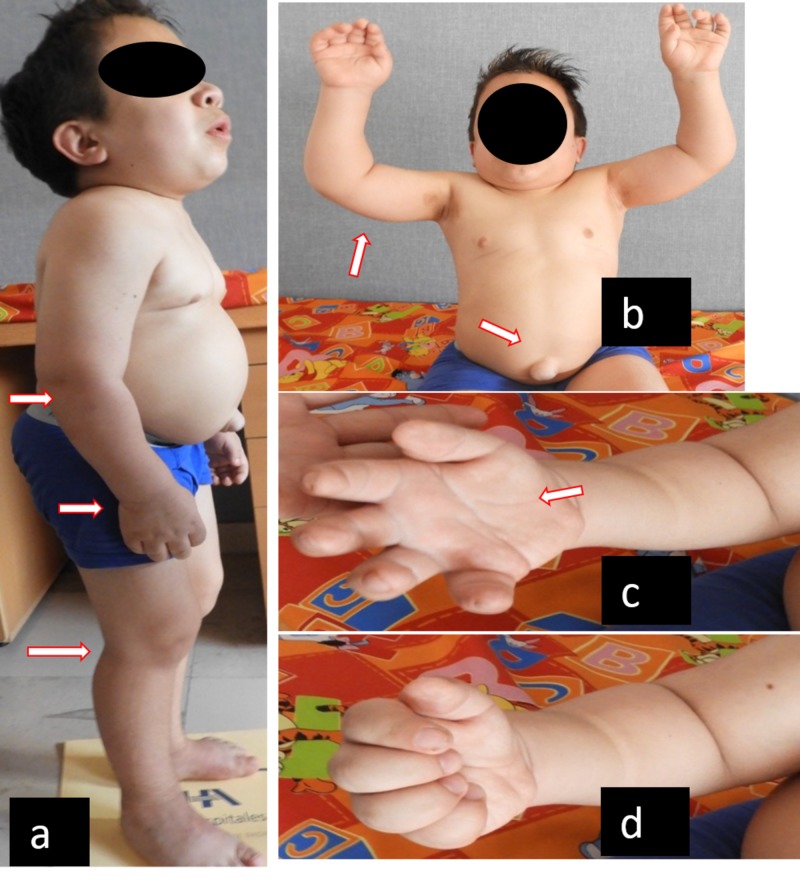
Clinical common characteristics in MPS patients a) Semiflexion contracture in elbows, wrists, hips, knees, and ankles. b) Contracture flexion in shoulders, incapacity to raise hands above the head, umbilical hernia. c) and d) Maximum active range of hand mobility with loss of grip force and hand skills. (This picture shows an MPS VI male patient). MPS: mucopolysaccharidosis

Some radiological findings expected in MPS include odontoid hypoplasia, atlantoaxial dislocation, J-shaped sella turcica, flattening of the ribs, Pectus carinatum, small iliacus bone, acetabular dysplasia, flattened capital femoral epiphysis, coxa plana, platyspondyly, central beaking of the first lumbar vertebrae and posterior vertebral scalloping, short ulna, and carpal and metacarpal bone dysplasia [2,4,10-11,13,15,18,20-21], as shown in Figure 2.

To confirm the diagnosis in a patient with suspected MPS, a dry peripheral blood spot test is taken on filter paper, determining the activity of the deficient enzyme. If the result is positive, it must be confirmed with the measurement of the enzyme's serum activity or mutational molecular analysis. Often, a mutational analysis is not routine and is only used for genetic counseling or research purposes [22].

Early diagnosis is important for prompt, specific treatment with enzyme replacement therapy (ERT), which is currently available for types I, II, IV and VI. Early start of treatment helps reduce the accumulation of intracellular GAGs and the deceleration of progressive multiorgan worsening, improving lung and heart function and final growth rate [1,23-25].

MPS are grouped into rare or orphan diseases. The main differential diagnosis is skeletal dysplasias [12,26]. The incidence varies upon the population studied and the type of MPS described; for example, for MPS IV A, the incidence ranges from 1:640000 live births in Australia to 1:76000 in Northern Ireland [13]. In Mexico, the diagnosis is confirmed around 4.5 years, usually by the geneticist or orthopedic surgeon [27].

Scarpa and collaborators reported in 2011, a European incidence from 1:140000 to 1:156000 live newborns for the MPS II [15].

Regard MPS VI, in 2007, an incidence from 1:238095 to 1:300000 live newborns were reported [14].

There is scarce literature addressing the common characteristics of these diseases and giving the clinician strong tools for a diagnostic suspicion. Diffusion will improve early diagnosis or refine diagnosis in patients with similar diseases.

The aim of the present report is to describe the clinical-radiographic features of patients with mucopolysaccharidosis in hospitals of the health system of Guanajuato.

## Materials and methods

In this retrospective study, all patients with a clinical diagnosis of mucopolysaccharidosis, who had confirmation of the enzymatic activity in the plasma, were included in the study, regardless of whether they were undergoing enzymatic replacement therapy (ERT) or not, in the hospitals of the Guanajuato Health System, including the Bajío High Specialty Regional Hospital, León General Hospital, Leon Pediatric Specialties Hospital, and General of San Hospital Luis de la Paz, in a period from January to December 2016. Those patients who were lost to follow-up or incomplete records were eliminated.

By direct clinical examination and data from the files, somatometry (weight, height, and body mass index) was obtained, as well as MPS features (corneal opacity, hernias, respiratory infections, joint movement, and orthopedic manifestations) since the first visit (before the diagnosis) and data that would lead to a suspicion of diagnosis.

Simple radiographs were used: pelvis posteroanterior, skull lateral, both knees posterior-anterior, hand anteroposterior, and lateral spine. We collected the main findings on electronic data sheets and an analysis was carried out by computer software.

Results were aggregated on Excel spreadsheets (Microsoft Corporation, Redmond, WA, US), formatted prior to analysis, and described with measures of central dispersion.

## Results

A total of 18 patients are reported on, Morquio A syndrome (MPS IV-A) predominates in seven patients (38%), followed by Hurler syndrome (MPS I) (22%) in four, San Filippo syndrome (MPS III) (16.6%) in three, and Hunter syndrome (MPS II) and Maroteaux-Lamie syndrome (MPS VI) in two cases each (11%, 11%, respectively).

Features at diagnosis

The first clinical manifestation related to the disease occurred at 3.1 years on average (range: birth to nine years). Earliest data were on intrauterine pneumonia in a patient with MPS I and the latest data were on knee valgus in a nine-year-old patient with MPS VI. The most commonly encountered alteration was genu valgus in 14 patients (77%) patients, of which four were severe (all MPS IV A), two moderate (all MPS VI), and the rest were mild. Other first manifestations were hernia (umbilical or inguinal) in five patients. Hernia together with infiltrated facies was the first finding in two patients and in three patients, hernia was the only first suspicion data. In three patients, the first manifestation was deformity in the thoracic cage (kyphosis or scoliosis). The rest of the patients showed limitations in joint mobility (rigidity), flat feet, and short stature, as shown in Table 2.

**Table 2:** Physical characteristics of present study MPS population MPS: type of mucopolysaccharidosis. H: umbilical or inguinal hernia. CO: corneal opacity. D: dimorphism. DD: dental diastasis. M: macroglossia. TD: thoracic deformity. HS: hepatosplenomegaly. LL: ligamentous laxity. DH: dorsal hump. GV: genu valgus. FF: flat feet

Patient	MPS	H	CO	D	DD	M	TD	HS	LL	DH	GV	FF
1	IV-A		●	●	●	●	●	●	●	●	●	●
2	IV-A		●	●	●	●	●	●	●	●	●	●
3	IV-A			●	●	●	●		●	●	●	●
4	IV-A	●		●	●	●	●		●	●	●	●
5	II			●		●		●				
6	III	●		●		●		●				
7	III	●		●	●	●		●				
8	IV-A		●	●	●	●	●	●	●	●	●	●
9	IV-A	●	●	●	●	●	●	●	●	●	●	●
10	VI	●	●	●	●	●	●	●		●	●	●
11	VI		●	●	●	●	●	●		●	●	●
12	IV-A	●		●	●	●	●	●		●	●	●
13	I	●	●	●	●	●	●	●		●	●	●
14	II	●		●	●	●	●			●		●
15	III	●		●	●	●		●		●	●	●
16	I	●	●	●	●	●	●	●		●	●	●
17	I	●	●	●	●	●	●	●	●	●	●	●
18	I	●	●	●	●	●	●	●		●	●	●

The time between the first observed manifestation and accurate diagnosis ranged between one month and six years (with an average of 2.7 years). The average age at diagnosis was 5.6 years (range from one month to 15 years).

All patients but one had a history of frequent respiratory infections (more than six events per year). Two of the patients died of complications thereof.

Half of the patients had a family history of this type of diseases, and six of the patients (33%) had consanguineous parents.

Physical features

All patients have dimorphism (infiltration) in variable ranges and relative macroglossia. Except for two patients (who died during the study) and one with MPS II, all of them showed a triangular dorsal hump. Other physical characteristics were hepatosplenomegaly in 15 patients, flat feet in 15 patients (88.3%), and genu valgus in 14 patients (ranging from mild to severe angulation). Hernia (inguinal or umbilical) was observed in 12 patients (66%). Fifty-five percent showed corneal opacity, 88.88% dental diastasis, and 100% relative macroglosia.

Radiographic studies showed 15 patients (83.33%) shared at least two or more characteristics, such as odontoid hypoplasia, atlantoaxial subluxation, J-shaped sella, widening of anterior ribs, pectus carinatum, flared ilium laterally with inferior constriction (wineglass shape), acetabular dysplasia, poorly formed femoral epiphyses, widened femoral necks, coxa valga, platispondilia, hypoplastic vertebra at the thoracolumbar junction, anterior inferior vertebral body beaking, short ulna, conical (bullet-shaped) proximal bases of metacarpals.

All patients with MPS III (n = 3) did not show any radiological alteration.

All patients with MPS IV A have undergone orthopedic surgical procedures at the time of the study.

## Discussion

Currently, seven types of MPS are known, caused by the deficiency of 10 hydrolases [8-9,22]. Within our study, only five types were presented, with a higher frequency of MPS IV A because the small sample size is not representative of the population, nor as an average to calculate the incidence. For example, in Western Australia, the general incidence of MPS is calculated as one in 29,000 live births, whereas the incidence of MPS III is the highest (one in 58000), followed by MPS I and, less frequently, MPS IV A (one in 640000) [28]; however, in Switzerland and Japan, the general incidence is calculated as 1.56 per 100000 live births; The first place in incidence corresponds to MPS. II (55%), followed by I, III, and IV A (12%, 24%, and 24%) [29]. A Pakistani report states that MPS I dominates in more than 80% of patients, followed by MPS IV A and III. The different data suggest that the marked difference in incidence depends on many factors, among which are consanguinity, cultural characteristics, and the underdiagnosis of these conditions [2,11-12].

The age of onset in more than 50% of patients with the severe form is 2.2 years, although the enzymatic diagnosis is usually delayed at least three years, which leads to a mean age for diagnosis of 4.9 years [11]. In the present report, the first manifestation was presented at 3.15 years with a diagnosis delay of 2.45 years average and age at enzymatic diagnosis was 5.6 years. On average for the Mexican MPS IV A population, the diagnosis is at 4.5 years in severe cases, like the present report, where the average age is five years [27].

Among the most common historical antecedents in these patients were: frequent respiratory infections, affected relatives, and consanguinity, as well as outstanding clinical characteristics, such as dental diastasis, macroglossia, hepatosplenomegaly, and infiltrated facies, in the same way as described by other publications. [2,6,17,21-22].

Orthopedic manifestations may be the first to be described by parents or guardians. In the present report, 11 of the 18 showed alterations of this kind, with genu valgus as the most commonly referred to; this agreed with the literature [3,18-19].

About 83.33% of the patients studied showed radiological characteristics like those reported in the literature [18,26].

Each type of Mucopolysaccharidosis has predominant, specific deposition organs. In case of MPS III, enzyme deficiency in the production of heparan sulfate has repercussions with the development of neurological symptoms, without skeletal muscle involvement as observed in the present series, where three patients with this disease did not present radiological data [12,16]. The severity of clinical characteristics seems to determine early or late diagnosis [30].

In the present report, the diagnosis was suspected on clinical and radiological findings, followed by filter paper screening and a confirmatory exam in the enzymatic test, according to the guidelines of international literature [8,22].

## Conclusions

Commonest clinical manifestations for diagnostic suspicion were dimorphism, skeletal alterations, triangular dorsal hump, genu valgus, restriction to mayor joint movement, and pes planus. Other frequent manifestations were short stature and umbilical or inguinal hernia. In the majority of the patients with mucopolysaccharidosis, radiological data of the disease were found, except for MPS III. The diagnosis was made by clinical and radiological evaluation and confirmed by an enzymatic study.

In all rare diseases, the diagnosis of suspicion is based on subtle characteristics that manifest themselves in different organs and systems. Suspicion by the physician and the need to strengthen collaboration patterns between different specialties play an important role in the early diagnosis and treatment of these conditions.

Greater familiarity with these diseases is necessary in first-contact physicians in order to improve the detection and decrease the complications of disease progression. It is a study whose main limitation is the small size of the sample, which only represents patients diagnosed in a specific geographical area of the center of a country.
